# Constructing the novel ultrafine amorphous iron oxyhydroxide/g-C_3_N_4_ nanosheets heterojunctions for highly improved photocatalytic performance

**DOI:** 10.1038/s41598-017-09283-1

**Published:** 2017-08-17

**Authors:** Hongcen Yang, Shouwei Zhang, Ruya Cao, Xiaolong Deng, Zhipeng Li, Xijin Xu

**Affiliations:** School of Physics and Technology, University of Jinan, Shandong, 250022 PR China

## Abstract

Ultrafine particles, more heterojunction interfaces and amorphous materials can effectively enhance the photocatalytic activity of photocatalysts. In this work, a facile *in-situ* precipitation method was developed to prepare ultrafine amorphous iron oxyhydroxide/ultrathin g-C_3_N_4_ nanosheets heterojunction composites. The amorphous iron oxyhydroxide possessed an ultrafine particle size and a wide range of visible light absorption. In this process, the ultrafine particles not only shortened the diffusion distance of photogenerated carriers, but also facilitated the formation of more heterojunctions with ultrathin g-C_3_N_4_ nanosheets. The photocatalytic activities were evaluated using rhodamine B, methylene blue, and methyl orange as pollution models under visible light irradiation. Notably, the optimal photocatalytic activity of a-FeOOH/CNNS-800 composite is ~17.8 times higher than that of CNNS towards the degradation of rhodamine B under visible light. The outstanding photocatalytic activities were ascribed to the narrower band gap, the enhanced visible light absorbance, abundant heterojunction interfaces, and the effective separation of the photogenerated charges driven by the matched band edge in the heterostructures. We trusted that the facile and easy-to-extend synthesis method can be further expanded to synthesize other ultrafine semiconductors coupled with g-C_3_N_4_ for enhancing the photocatalytic activities.

## Introduction

In recent years, the environmental pollution is becoming more and more serious with the development of economy, which has become a serious threat to human survival and development^1–4^. The semiconductor photocatalytic technique has been regarded as an important way to solve this issue by decomposing toxic and hazardous organic pollutants into non-toxic products^5–7^. However, the traditional semiconductor photocatalytic technique has some drawbacks, such as the only absorb UV light and the low photocatalytic efficiencies^3, 8–10^. Therefore, the development of novel photocatalysts with visible-light response and high photocatalytic efficiencies has become urgent^8, 11, 12^.

Graphite carbon nitride (g-C_3_N_4_), as metal-free semiconductor, has received great attention in photocatalytic degradation of organic pollutants, due to its unique three-dimensional layered structure, relatively narrow band gap (~2.7 eV) and low cost^13–16^. However, a low quantum efficiency and narrow visible-light response range restricted its application^5, 17^. To conquer these defects, numerous strategies have been explored, including combination with carbon materials, metal and/or non-metallic materials doping, formation of heterojunctions, and so on^9, 12, 18, 19^. For example, a series of g-C_3_N_4_-based photocatalysts was constructed by Zhang group and G. Mamba group, which enhanced the visible-light photocatalytic performance through the formation of heterojunctions^4, 20^. Among these, the coupling of other semiconductors with suitable bandgap on g-C_3_N_4_ to form heterojunctions is an effective method to enhance visible-light absorption ranges and improve photogenerated charge separation and transfer^18^. However, it is still a challenge to explore suitable semiconductors to satisfy these requirements.

The amorphous iron oxyhydroxide (a-FeOOH), as one kind of iron oxides/hydroxides, has attracted attention for hydrogen production from water splitting and degradation of toxic organic pollutants under UV or visible-light irradiation^21–23^. The amorphous iron oxyhydroxide is easy to get in the natural environment with the features of non-toxic, corrosion resistant and low cost^19, 23–26^. In addition, the narrow band gap endows wide light response ranges to ensure harvest visible light, which is the premise to achieve high photocatalytic efficiency^20, 25, 27, 28^. Accordingly, it is expected that the amorphous iron oxyhydroxide coupled g-C_3_N_4_ would show high photocatalytic performance due to the following reasons: (i) it could obtain a wide range of visible light absorption; (ii) it has a suitable band structure, which could form heterojunctions, decreased the recombination of photogenerated charge and improved the photocatalytic efficiency^17, 29^.

As is known to all, interfacial charge transfer between the discrete energy levels of molecules and the continuous energy levels of solids has been an effective method to raise the photocatalytic activity of semiconductor photocatalysts^6, 30^. Furthermore, abundant heterojunction interface will separate the photogenerated electron-hole pairs more effectively^18, 31^. Therefore, it is feasible to enhance the photocatalytic activities of g-C_3_N_4_-based photocatalysts by tuning the size and distribution of the coupled nanoparticles benefited from the two merits: (i) the smaller particles obviously shorten the diffusion distance of photogenerated carriers, improving the photocatalytic performance; (ii) ultrafine particles coupling with g-C_3_N_4_ can generate more effective heterojunctions and further facilitate the synergistic reaction between semiconductors and g-C_3_N_4_^32–35^. In other words, ultrafine nanoparticles and more efficient heterojunctions can also enhance the catalytic properties of photocatalysts^32–35^.

In this work, we prepared the novel ultrafine amorphous iron oxyhydroxide/g-C_3_N_4_ nanosheets heterojunction nanocomposites through a simple *in-situ* precipitation method by uniformly dispersing ultrafine amorphous iron oxyhydroxide to the surface of g-C_3_N_4_ nanosheets. During the reaction, the designed amorphous iron oxyhydroxide/g-C_3_N_4_ nanosheets heterostructures are featured with an ultrafine amorphous iron oxyhydroxide anchored on the ultrathin g-C_3_N_4_ nanosheets, providing a large surface area and abundant heterojunction interfaces. The photocatalytic activities of amorphous iron oxyhydroxide/g-C_3_N_4_ nanosheets were evaluated by degrading rhodamine B (RhB), methylene blue (MB) and methyl orange (MO) under visible light irradiation. In addition, the degradation mechanism was also proposed.

## Experimental sections

### Preparation of the amorphous iron oxyhydroxide (a-FeOOH)

a-FeOOH was synthesized by a simple *in-situ* precipitation method. Firstly, 1 mmol FeCl_3_·6H_2_O was dissolved in 150 mL anhydrous ethanol under stirring. Then, 3 mmol ammonium bicarbonate (NH_4_HCO_3_) was added with stirring, and the solution kept stirring for 8 hours. Finally, the precipitates were collected and washed with anhydrous ethanol, followed by vacuum freeze drying.

### Synthesis of g-C_3_N_4_ nanosheets (CNNS)

Urea was placed in the covered crucible and heated under static air at 550 °C for 4 h with a ramp rate of 2.5 °C/min. The as-obtained powders were placed in an open crucible and further heated at 500 °C for 2 h with a ramp rate of 5 °C/min. After cooling down to room temperature, the desired ultrathin CNNS were obtained by washing with deionized water and further dried in a vacuum oven.

### Synthesis of amorphous iron oxyhydroxide/g-C_3_N_4_ nanosheets composites (a-FeOOH/CNNS)

The a-FeOOH/CNNS composites were synthesized by a facile *in-situ* precipitation method, similar as the synthesis of a-FeOOH. Specifically, 1 mmol FeCl_3_·6H_2_O was dissolved in 150 mL of anhydrous ethanol. Then, a certain amount of CNNS were added into the solution and sonicated for 2 h to disperse CNNS. After that, 3 mmol ammonium bicarbonate (NH_4_HCO_3_) was added into the suspension and the reaction was continued another 8 hours. The precipitates were collected and washed with anhydrous ethanol, followed by vacuum freeze drying. a-FeOOH/CNNS composites prepared with different amounts of CNNS in 300, 400, 500, 600, 700, 800 and 900 mg were denominated as a-FeOOH/CNNS-300, a-FeOOH/CNNS-400, a-FeOOH/CNNS-500, a-FeOOH/CNNS-600, a-FeOOH/CNNS-700, a-FeOOH/CNNS-800 and a-FeOOH/CNNS-900, respectively.

### Characterization

Powder X-ray diffraction (XRD) data were obtained via a D/MAX2500 V diffractometer equipped with Cu Kα radiation (λ = 1.5418 Å). The infrared absorption spectra of the materials were measured by a Fourier transform spectrophotometer (FT-IR, Avatar 370, Thermo Nicolet) using the standard KBr disk method. X-ray photoelectron spectroscopy (XPS) measurements were conducted on ESCALAB250 with Mg Kα as the source and the C 1s peak at 284.6 eV as an internal standard. The morphologies of the materials were observed using a FEI QUANTA FEG250 field emission scanning electron microscope (SEM) and a Tecnai G2 F20 S-TWIN transmission electron microscope (TEM) at an accelerating voltage of 200 kV as well as the composites. UV-vis diffuse reflection spectroscopy (DRS) was performed on a Shimadzu UV-2500 spectrophotometer using BaSO_4_ as the reference.

### Evaluation of photocatalytic activity

The photocatalytic activity of as-prepared catalysts was evaluated by degrading RhB, MB and MO under a 500 W Xe lamp equipped with a cutoff filter (λ ≥ 420 nm) as a light source. In short, 50 mg photocatalysts were added into 50 mL of RhB (or MB, MO) aqueous solution (10 mg/L). The mixed solution was magnetically stirred for 60 min to achieve adsorption–desorption equilibrium before turning on Xe lamp. During the photocatalytic test, 3 mL suspension was sampled, followed by centrifugation to separate the photocatalyst at a certain irradiation time interval, and the photocatalytic efficiency was tested by a UV-vis spectro-photometer (UV-2500, Shimadzu).

## Results and Discussion

**Figure 1:**
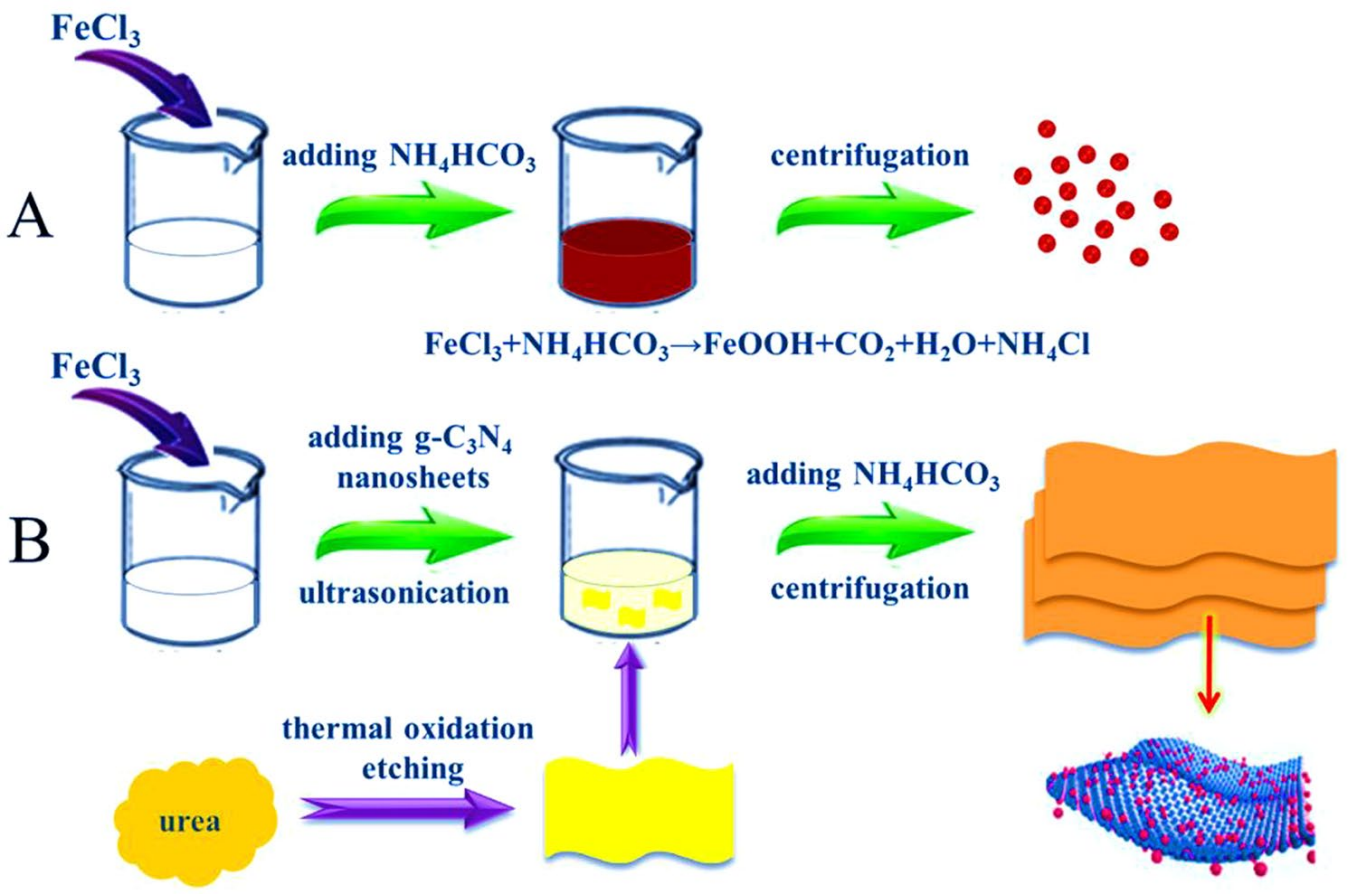
Schematic illustration of the synthesis of (**A**) a-FeOOH and (**B**) a-FeOOH/CNNS composites.

A simple *in-situ* precipitation synthesis method was designed to prepare a-FeOOH and a-FeOOH/CNNS hybrid hierarchical three-dimensional architectures, repesctively, and the preparation process was schematically shown in Fig. 1. The process may be described as follows^22^:

$${{\rm{FeCl}}}_{3}+{{\rm{NH}}}_{4}{{\rm{HCO}}}_{3}\to {\rm{FeOOH}}+{{\rm{CO}}}_{2}+{{\rm{H}}}_{2}{\rm{O}}+{{\rm{NH}}}_{4}{\rm{Cl}}$$

For the preparation of a-FeOOH, NH_4_HCO_3_ was added to the FeCl_3_ solution with the formation of a-FeOOH. The chemical reaction was carried out very homogeneously forming ultrafine a-FeOOH nanoparticles. CNNS could be easily obtained by thermal oxidation etching of bulk g-C_3_N_4_ in air. The dispersed CNNS were negatively charged, with a zeta potential of about −27.6 mV (Figure S1). By adding CNNS into FeCl_3_ solution, Fe^3+^ cations would be bound tightly to the surface of CNNS via electrostatic interactions. Then, with the addition of NH_4_HCO_3_, the fixed Fe^3+^ cations would further react with NH_4_HCO_3_ to generate a-FeOOH, resulting in a-FeOOH/CNNS. In this case, CNNS not only provided a large surface area, but also acted as the support to form heterostructures. This synthetic route to a-FeOOH/CNNS photocatalysts was economic, facile and could be produced in a large scale, which provided new opportunities for the preparation of amorphous metal oxide nanoparticles.

**Figure 2:**
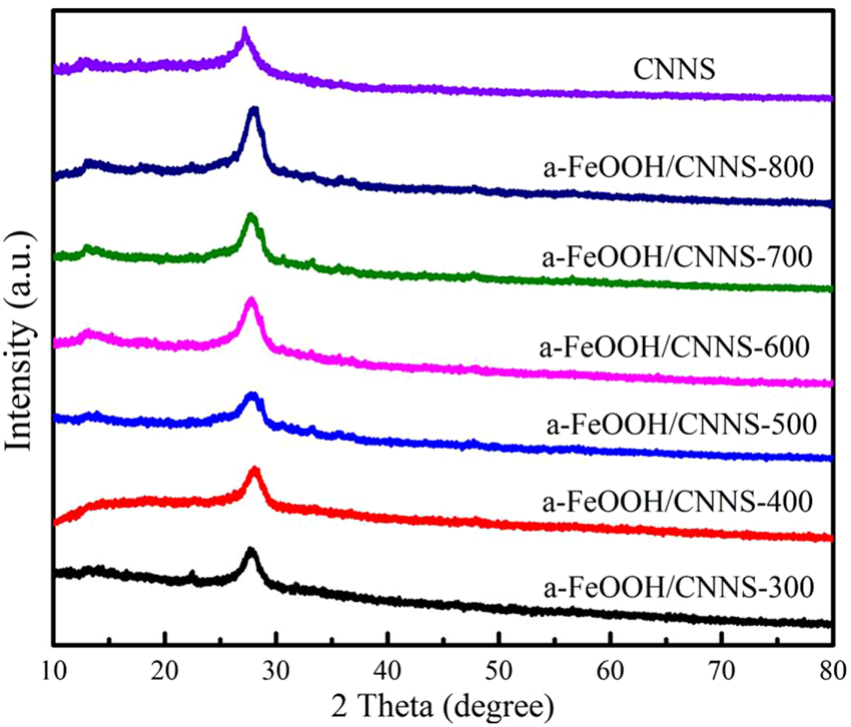
XRD patterns of CNNS and a-FeOOH/CNNS composites.

Figure 2 showed the XRD patterns of pure CNNS and a-FeOOH/CNNS composites with different amounts of CNNS. For pure CNNS, the diffraction peaks at 13.1° and 27.8° corresponded to (100) and (002) diffraction planes of graphitic carbon nitride, respectively^3, 7, 18, 36^. All XRD patterns of composites did not show the diffraction peaks of a-FeOOH, probably indicating that the amorphous feature of a-FeOOH or the few content of a-FeOOH in the composites^22^.

**Figure 3:**
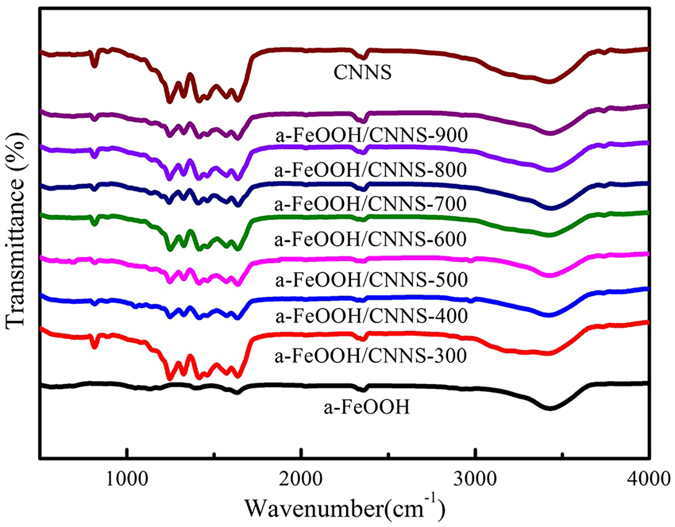
FT-IR spectra of CNNS, a-FeOOH and a-FeOOH/CNNS composites.

Figure 3 exhibited the FT-IR spectra of pure CNNS and a-FeOOH/CNNS composites, which was used to reflect the difference in chemical structure of materials. For pure CNNS and its composites, the broadened band in the range of 3000–3700 cm^−1^ represented the N-H stretching vibration of CNNS^1, 3, 6^. The several strong peaks within 1400–1800 cm^−1^ belonged to C = N and C-N heterocycles skeletal vibration of the aromatic ring^7–9^. The peaks at 1245 and 1325 cm^−1^ indicated the stretching vibrations of C-NH-C bridges^6–9^. And the sharp peak at 814 cm^−1^ was in line with the C-N stretching vibration of the feature of the triazine cycles, which confirmed the successful introduction of CNNS in the composites^9, 11, 12^. A small peak at 2356 cm^−1^ was attributed to the appearance of C≡N and N = C = N, which might be from the small fragment of diazo groups adhered to the surface, but in fact these may not have an impact on the formation of this organic network structure^7, 8^. For a-FeOOH/CNNS composites, no obvious differences were observed in comparing with CNNS, probably manifesting the introduction of a-FeOOH did not change the chemical structure of CNNS or the content of a-FeOOH was very small^1, 6^. So XPS was used to demonstrate the existence of a-FeOOH.

**Figure 4:**
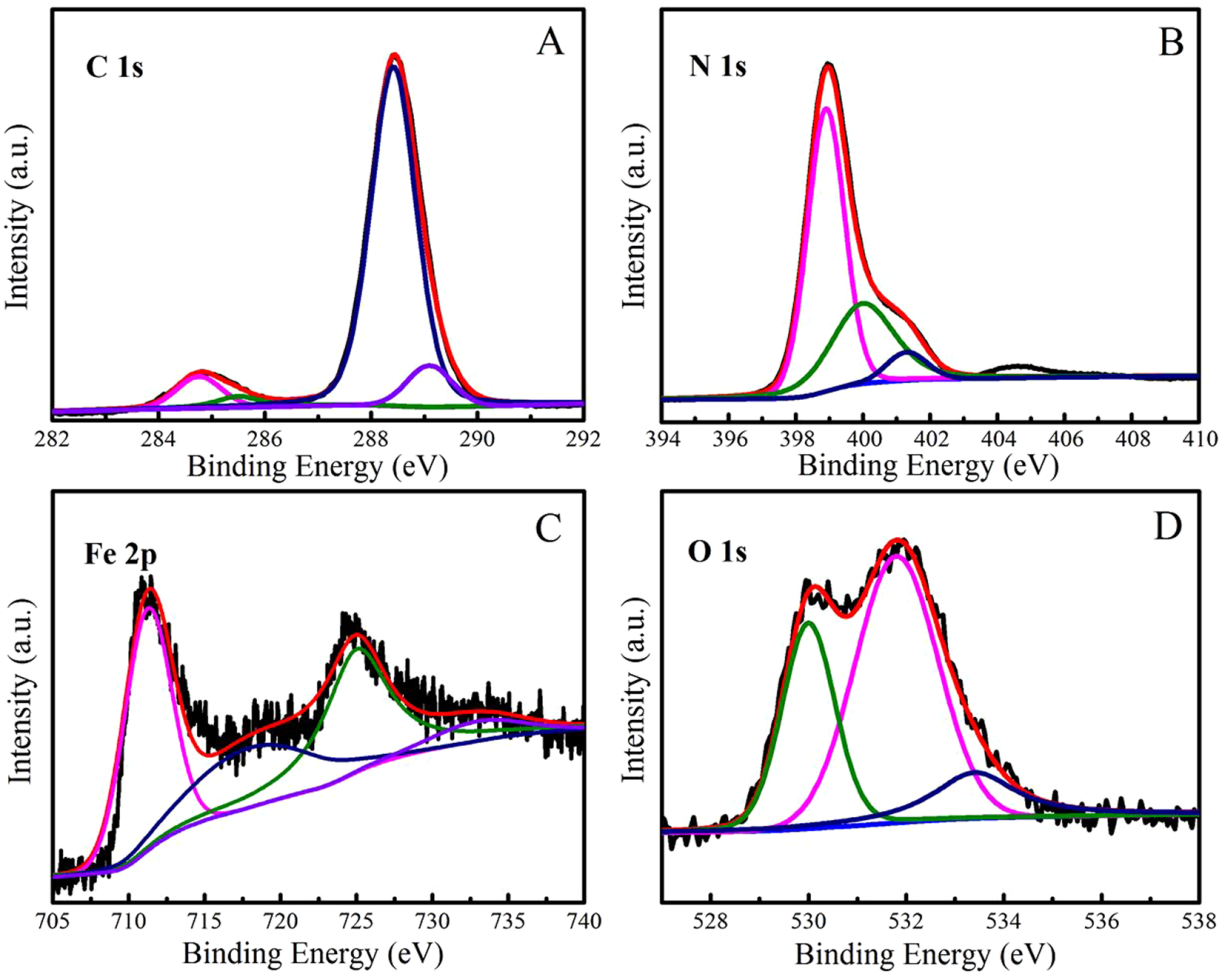
(**A**) and (**B**) high resolution spectra of C 1s and N 1s for a-FeOOH/CNNS-800 composite; (**C**) and (**D**) high resolution spectra of Fe 2p and O 1s for a-FeOOH/CNNS-800 composite.

The elements of the synthesized a-FeOOH/CNNS-800 by the XPS spectra were shown in Fig. 4. The XPS spectra demonstrated the presence of C, N, Fe and O in a-FeOOH/CNNS-800 hybrid (Figure S2). In the C 1s spectra of a-FeOOH/CNNS-800 (Fig. 4A), the C 1s peak with a binding energy of 284.75 eV was considered to be the standard reference carbon from the background^5, 6, 37^. In addition to the standard reference carbon, the high resolution C 1s spectrum was also fitted into three carbonaceous species with different binding energy^38^. The main peak with a binding energy of 288.41 eV represented sp^3^-C = N bonds, which made up the main structure of CNNS^18, 37, 38^. There were two small peaks with the binding energy of 285.49 and 289.09 eV. The peak with a binding energy of 285.49 eV could be explained as surface hydroxyl (C-OH) bond, while the peak with a binding energy of 289.09 eV was identified as carbonyl (O-C = O) bond^8, 9^. These two peaks were often used as the evidence of successful surface modification^6^.

For the N 1s spectrum (Fig. 4B), there displayed three nitrogen states. The strong peak at 398.89 eV could be attributed to sp^2^-hybridizednitrogen (C = N-C) coordination in CNNS structures^5, 6, 39^. The peak locating at 399.99 eV indicated tertiary nitrogen (N-(C)_3_) groups (quaternary N), while the peak with a binding energy of 401.29 eV was assigned to amino functions (C-N-H)^6, 7^. These states corresponded to three nitrogen units that constituted the basic units of CNNS^17, 18^. The weak peak with a binding energy of 404.60 eV was attributed to the charging effects or positive charge localization in the heterocycles^38^. It meant that the nanostructure of CNNS was not changed after it has been compounded with a-FeOOH^20^. The Fe 2p spectrum (Fig. 4C) depicted four peaks with binding energy of 710.90, 725.01, 718.04 and 733.02 eV. The formation of a-FeOOH was proven by two major peaks located at 710.93 eV for Fe 2p_3/2_ and 725.06 eV for Fe 2p_1/2_, which corresponded to Fe^3+^.^40, 41^ Shake-up satellite peaks of Fe 2p_3/2_ and Fe 2p_1/2_ can also be observed around 718.02 and 733.07 eV^42–44^. In the O 1s spectrum (Fig. 4D), the peaks located at 530.05 and 531.80 eV were assigned to Fe-O-Fe bond and Fe-O-H bond, respectively, while the weak peak at 533.40 eV was assigned to H-O-H bond^38, 40^.The discovery of Fe-O-Fe and Fe-O-H bonds also demonstrated the presence of a-FeOOH^22^.

**Figure 5:**
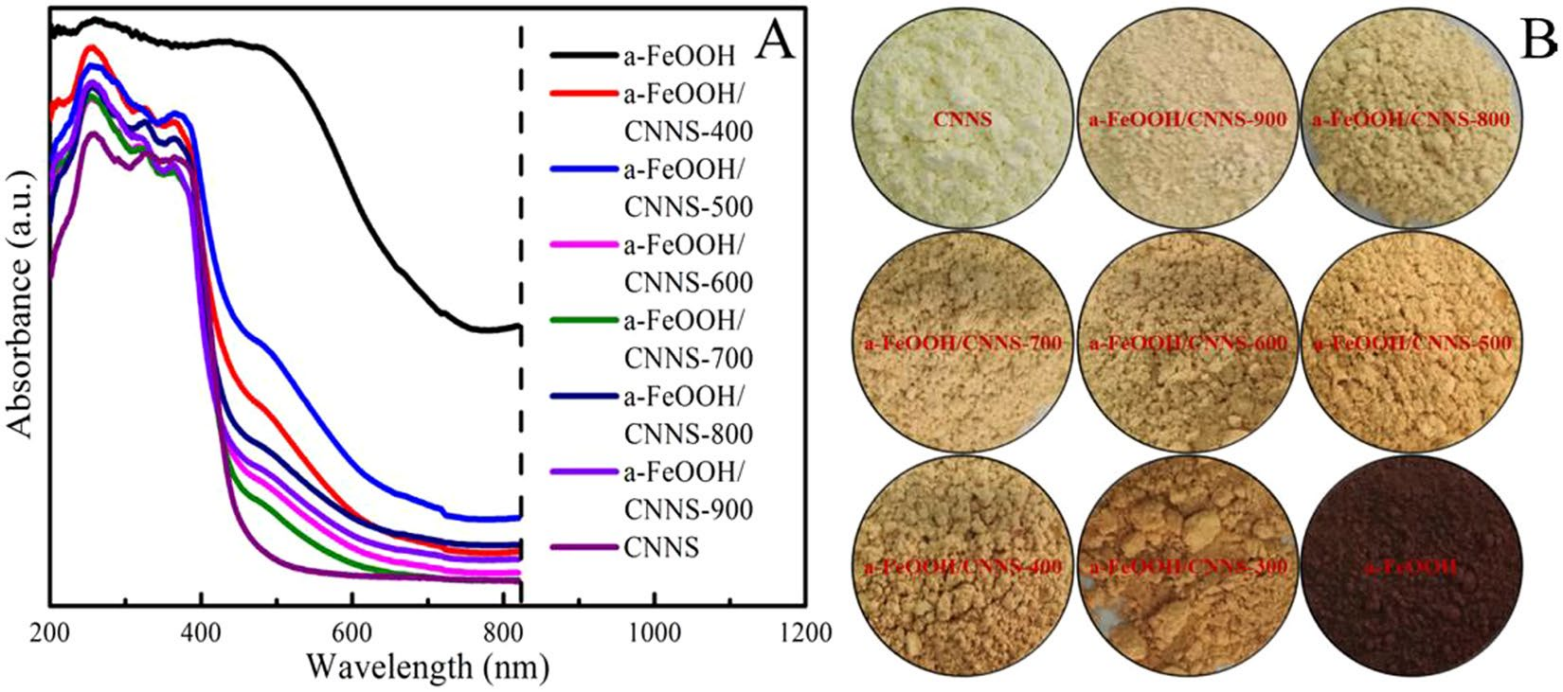
(**A**) UV-vis diffuse reflectance spectra and (**B**) digital photographs of CNNS, a-FeOOH and a-FeOOH/CNNS composites.

Figure 5A indicated the light absorption properties of CNNS, a-FeOOH and a-FeOOH/CNNS by UV-vis DRS. The light absorption edge of CNNS was ~454 nm, corresponded to a band gap of ~2.73 eV, which was slightly blue shift relative to the band gap of the bulk g-C_3_N_4_, this indicated that CNNS has thinner nanosheets^18^. The light absorption edge of a-FeOOH was more than 800 nm, so a-FeOOH can absorbed nearly all visible lights. The a-FeOOH/CNNS composites presented the hybrid absorption features of both a-FeOOH and CNNS. Compared with CNNS, all a-FeOOH/CNNS composites exhibited broader visible light absorptions due to intimate interfacial contact between a-FeOOH and CNNS, resulted in the change of optical properties of the composites^6^. The strength of the absorption bands enhanced with the quantity increase of a-FeOOH corresponding to the color change of the composites (Fig. 5B), which turned from light yellow to reddish brown with the increase of a-FeOOH. It is deduced that the light absorption abilities of CNNS could be significantly enhanced through heterojunctions by way of loading a-FeOOH as “nanometer island” on the surface of CNNS, resulting in a decrease in the interface contact barrier and an enhancement of the electron coupling of the semiconductor, which were beneficial to generate more photogenerated electrons/holes with improved photocatalytic performance^18^.

The energy levels and band gap of semiconductor photocatalysts play a crucial role in determining photocatalytic performance. The band gap energy (E_g_) of a semiconductor can be calculated by the following Eq.:

$${\rm{\alpha }}h{\rm{\nu }}={\rm{A}}{(h\nu -{{\rm{E}}}_{{\rm{g}}})}^{n/2}$$

where α, h, ν, E_g_ and A represent the light absorption coefficient, Planck constant, light frequency, band gap energy, and constant, respectively. Among them, n is determined by the type of optical transition of a semiconductor (n = 1 for direct transition and n = 4 for indirect transition). The band gaps are estimated to be 2.56 eV and 0.91 eV for CNNS and a-FeOOH respectively (Figure S3), according to a plot of (αhν)^1/2^ versus energy (hν).

**Figure 6:**
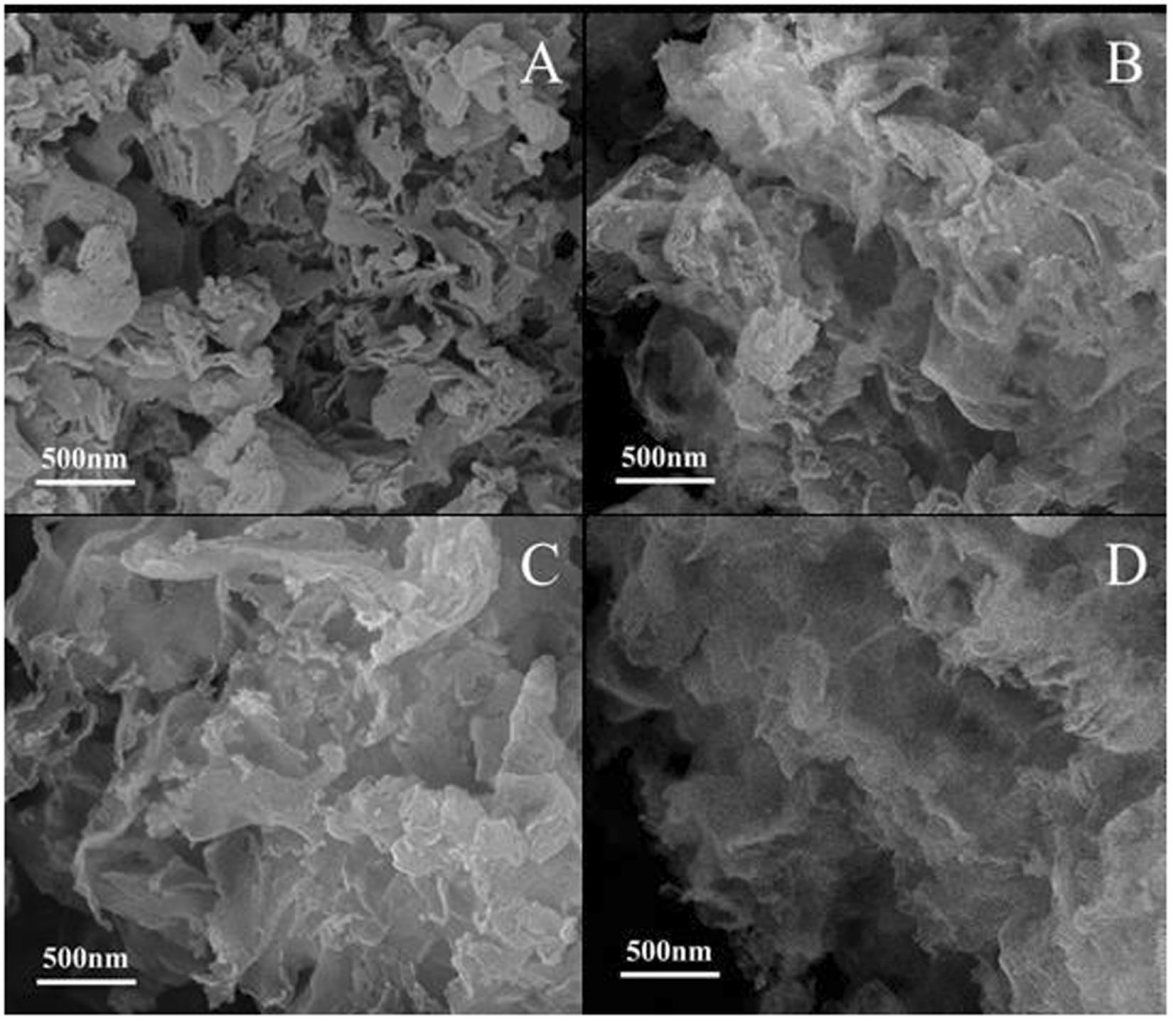
SEM images of (**A**) CNNS, (**B**) a-FeOOH/CNNS-400 composite, (**C**) a-FeOOH/CNNS-600 composite and (**D**) a-FeOOH/CNNS-800 composite.

**Figure 7:**
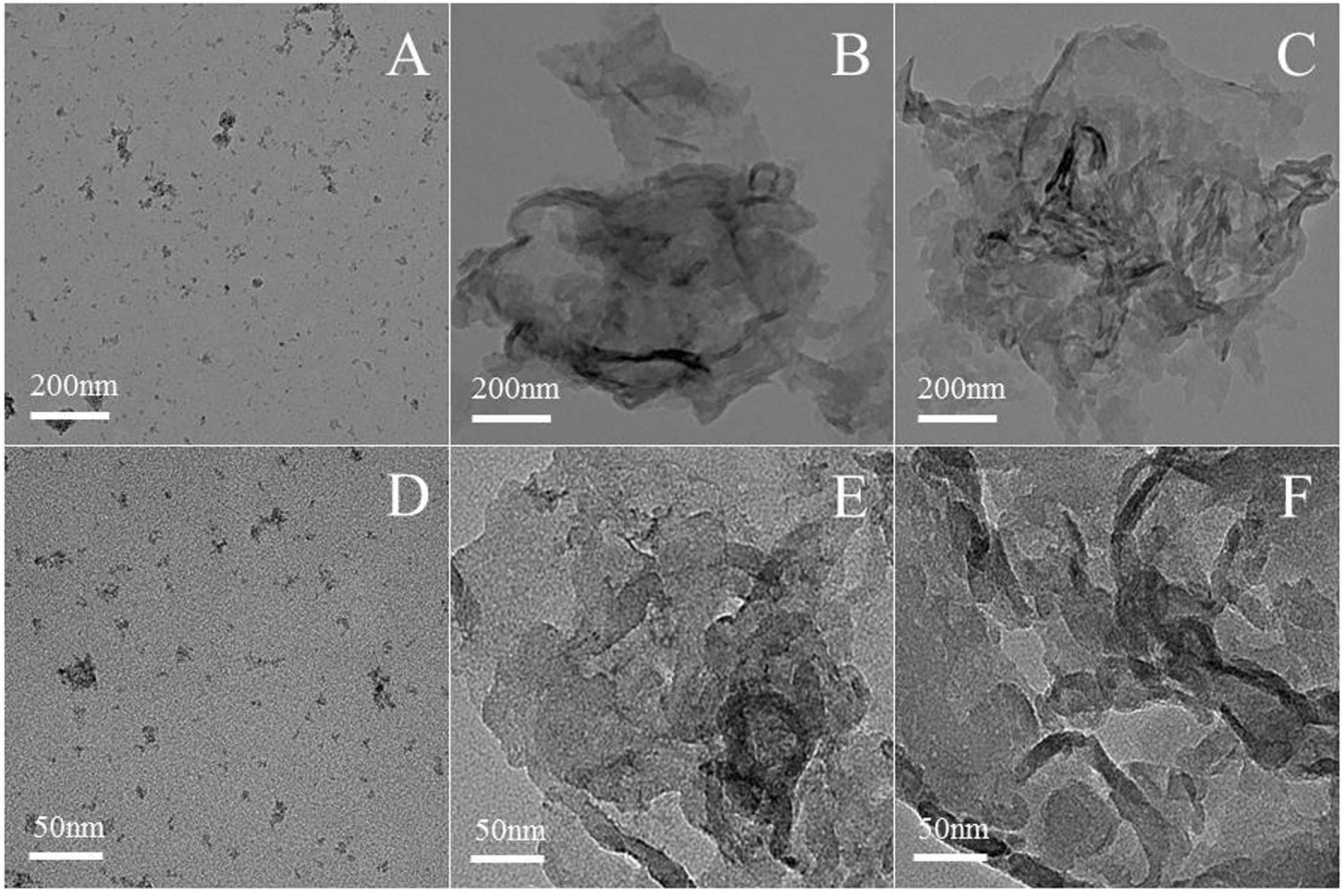
TEM images of (**A**) and (**D**) a-FeOOH, (**B**) and (**E**) a-FeOOH/CNNS-800 composite and (**C**) and (**F**) CNNS.

**Figure 8:**
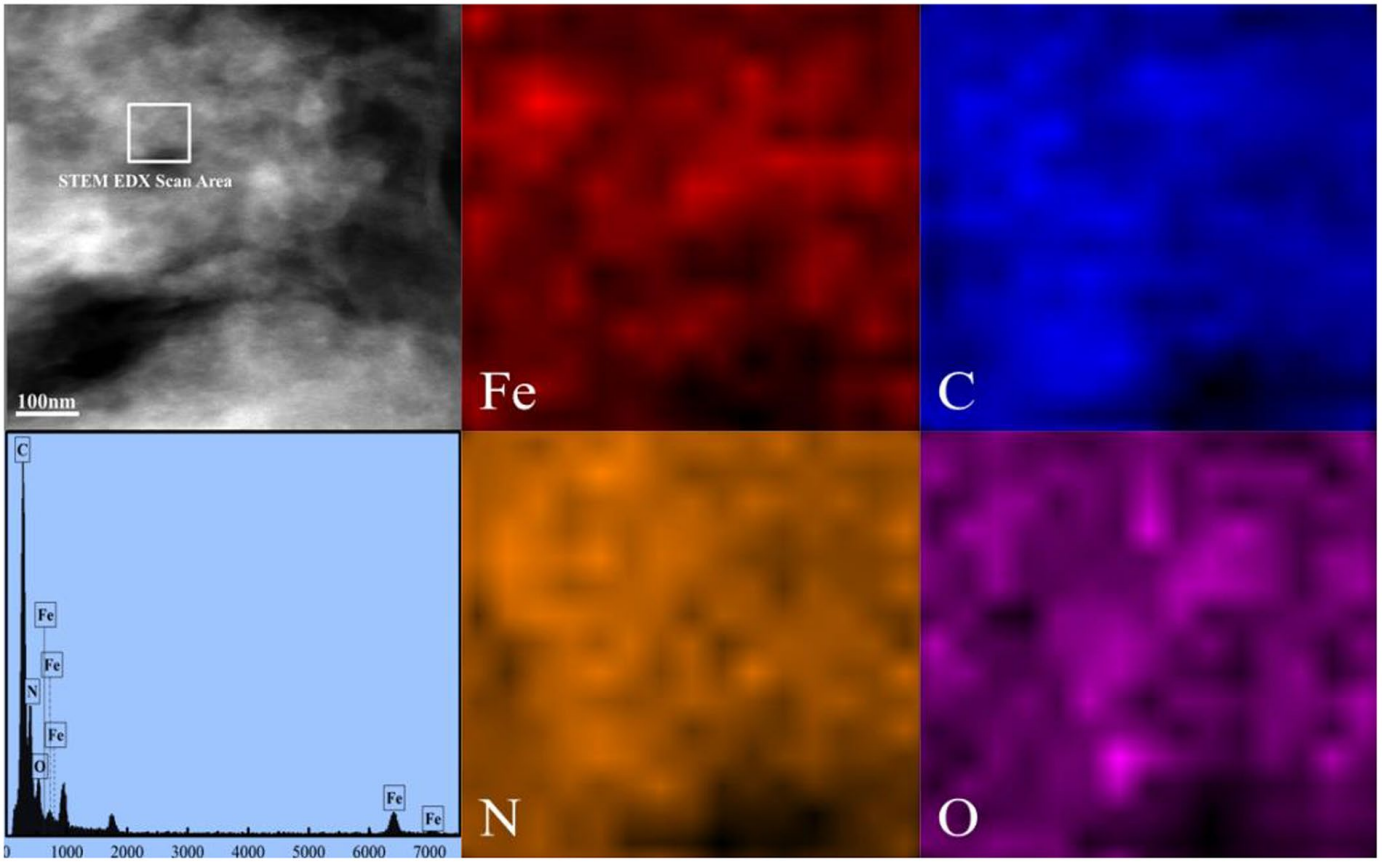
The elemental mapping images and EDX spectrum of the a-FeOOH/CNNS-800 composite.

The morphologies of a-FeOOH, a-FeOOH/CNNS-800 and CNNS were characterized using electron microscopes. The related SEM and TEM images are shown in Figs 6 and 7, respectively. It can be seen that CNNS, a-FeOOH/CNNS-400, a-FeOOH/CNNS-600 and a-FeOOH/CNNS-800 exhibit three-dimensional layered structures from the SEM images (Fig. 6), which was favorable for the increase of the reaction surface and surface active sites. As shown in Fig. 7A and D, the particle size of pure a-FeOOH is very small (1–8 nm). The a-FeOOH/CNNS-800 (Fig. 7B and E) and CNNS (Fig. 7C and F) were observed as thin nanosheets with wrinkles, in which overlapped layers were described as black stripes. The SEM and TEM images of CNNS and a-FeOOH/CNNS composites suggested that the morphology of a-FeOOH/CNNS composites did not change compared with pure CNNS. However, a-FeOOH cannot be differentiated from CNNS in the SEM and TEM images due to the ultrafine granularity, so Mapping and EDX was used to further detect the presence of a-FeOOH. The uniformly dispersing ultrafine a-FeOOH on the entire surface of CNNS could be further verified by the elemental mapping and EDS images (Fig. 8), where Fe, C, N and O elements were homogeneously distributed over the whole profile, demonstrating the successful coupling of a-FeOOH with CNNS to form heterostructures.

**Figure 9:**
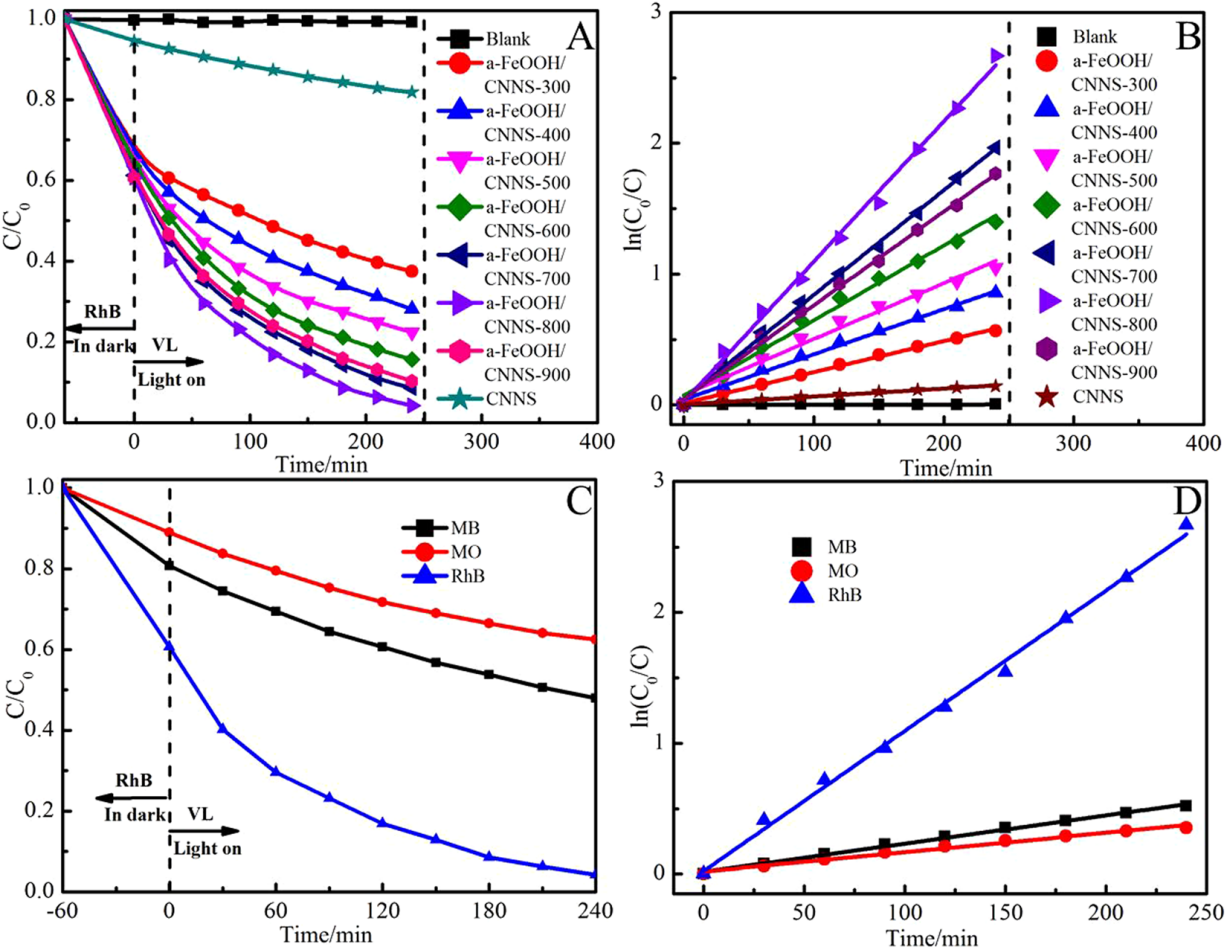
(**A**) The photocatalytic activities of the as-prepared photocatalysts for the degradation of RhB under visible light irradiation; (**B**) The rate constants of the as-prepared photocatalysts for the degradation of RhB; (**C**) The photocatalytic activities of the as-prepared photocatalysts for the degradation of RhB, MB, MO under visible light irradiation; (**D**) The rate constants of the as-prepared photocatalysts for the degradation of RhB, MB, MO.

**Figure 10:**
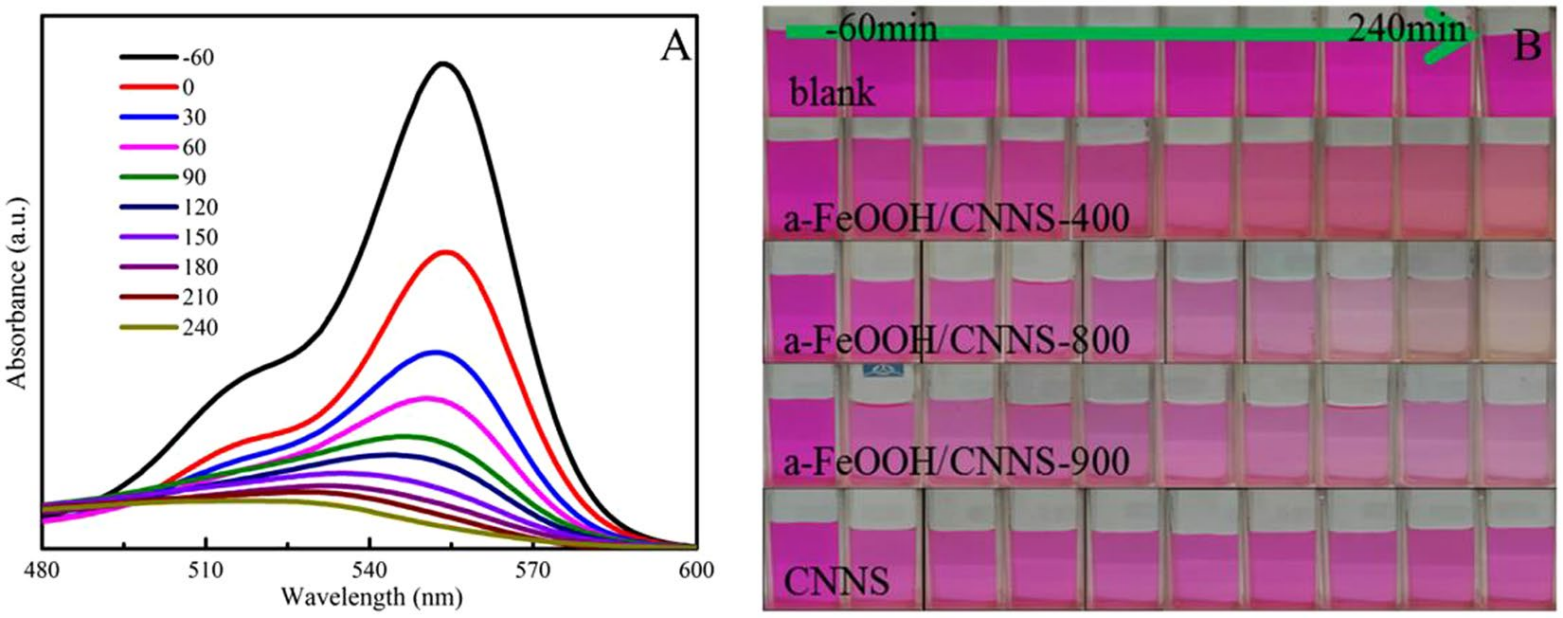
(**A**) The absorption spectra of RhB degraded by a-FeOOH/CNNS-800 composite under visible light irradiation; (**B**) The corresponding digital photograph of RhB degraded by a-FeOOH/CNNS composites under visible light irradiation.

To evaluate the photocatalytic activities of a-FeOOH/CNNS composites under visible light irradiation, RhB was chosen as model pollutant for photocatalytic degradation (Fig. 9A). The adsorption of RhB on a-FeOOH/CNNS in the darkness reached equilibrium within 60 min (Figure S4) and negligible self-degradation was also observed for RhB under visible light irradiation. As expected, all a-FeOOH/CNNS composites exhibited higher photocatalytic activities than either a-FeOOH or CNNS by visible light irradiation with a sequence of a-FeOOH/CNNS-800 > a-FeOOH/CNNS-700 > a-FeOOH/CNNS-900 > a-FeOOH/CNNS-600 > a-FeOOH/CNNS-500 > a-FeOOH/CNNS-400 > a-FeOOH/CNNS-300 > CNNS, indicating the positive effect of CNNS contents to promote the photocatalytic activities of a-FeOOH/CNNS composites. Full degradation of RhB was observed within 240 min by visible light irradiation in the presence of a-FeOOH/CNNS-800 composite, illustrating the significantly improved photocatalytic activity of the ultrafine a-FeOOH NPs/CNNS composites. However, further increment of CNNS (a-FeOOH/CNNS-900) resulted in decreased photocatalytic activity, which may be attributed to the recombination of photogenerated electrons and holes to restrain the photocatalytic efficiency. Figure 10A shows the variation of the absorption spectra of RhB under visible light irradiation by using a-FeOOH/CNNS-800. The characteristic peak intensities of RhB gradually decreased by prolonging the irradiation time, and the adsorption peaks disappeared within ~240 min irradiation. The maximum absorption wavelength edisplayed a blue shift, which indicated that the de-ethylation process^46^. These corresponding optical photographs of RhB degradation using different photocatalysts under different irradiation times were collected and displayed in Fig. 10B.

In order to further assess the mineralization of RhB in water, total organic carbon (TOC) was also monitored during the reaction process, and a-FeOOH/CNNS-800 was selected as the representative photocatalyst, and the result is shown in Figure S5. TOC of RhB is degraded by 55% after exposure to visible light irradiation for 240 min, indicating that the produced organic intermediates continue to decompose into inorganic species. It is verified that RhB is indeed photocatalytic degraded by a-FeOOH/CNNS-800 composite, rather than just decolorized by the light irradiation.

To investigate the photodegradation kinetics of RhB, the photodegradation data were further analyzed by the pseudo-first-order model. This analysis was deduced from the pseudo-first-order model in the following Eq.^12, 20^

$$\mathrm{ln}(\frac{{C}}{{{C}}_{{0}}})=-{Kt}$$

*K* (min^−1^) is the rate constant, *C*_0_ (mg/L) is the initial concentration of RhB, and *C* (mg/L) is the concentration of RhB at time *t* (min). The highest degradation rates for RhB (Fig. 9B) were calculated to be 0.0107 min^−1^ for a-FeOOH/CNNS-800, which were ~17.8, ~4.6, ~3.1, ~2.5, ~1.8, ~1.3 and ~1.5 times higher than those of CNNS (0.0006 min^−1^), a-FeOOH/CNNS-300 (0.0023 min^−1^), a-FeOOH/CNNS-400 (0.0034 min^−1^), a-FeOOH/CNNS-500 (0.0042 min^−1^), a-FeOOH/CNNS-600 (0.0057 min^−1^), a-FeOOH/CNNS-700 (0.008 min^−1^) and a-FeOOH/CNNS-900 (0.0071 min^−1^), respectively.

In order to test the photocatalytic activity of a-FeOOH/CNNS-800 for different pollutants (MO and MB), were chosen as model pollutants for photocatalytic degradation under visible light irradiation (Fig. 9C). As expected, a-FeOOH/CNNS-800 can also degrade MO (0.0015 min^−1^) and MB (0.0022 min^−1^) under visible light irradiation (Fig. 9D), indicating the positive effect of a-FeOOH to expand the universality of a-FeOOH/CNNS composites.

The excellent photocatalytic performance a-FeOOH/CNNS composites could be attributed to the synergistic interactions from the following aspects: (i) the ultrafine a-FeOOH NPs can shorten diffusion distance of photogenerated charge to the surface; (ii) for the synthesis of a-FeOOH/CNNS composites, CNNS is simply introduced into the reaction system and the a-FeOOH is tightly anchored by strong electrostatic interaction, which can ensure the stability of the nanocomposites; (iii) the three-dimensional layered structures can provide more reactive sites for the adsorption and degradation of organic pollutants^18, 45, 47^.

**Figure 11:**
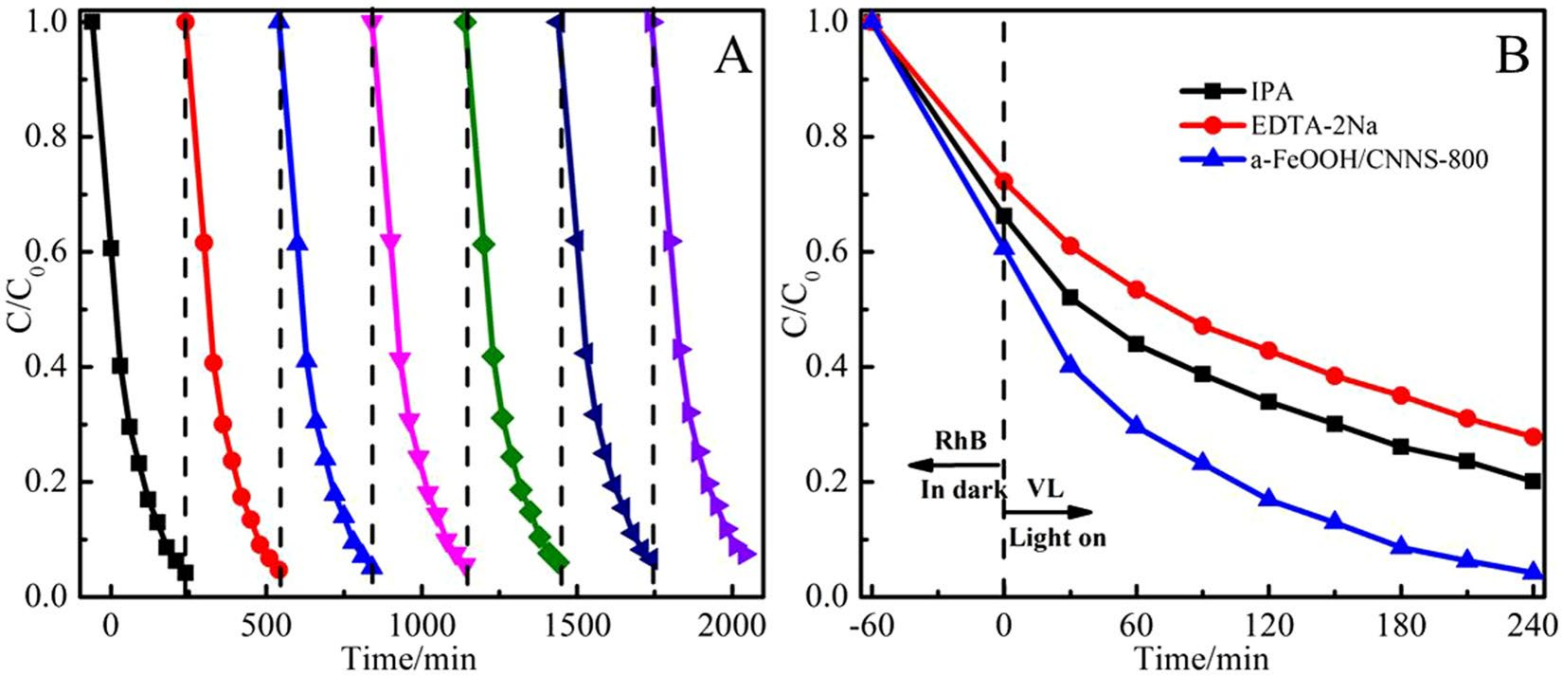
(**A**) Stability of a-FeOOH/CNNS-800 composite for RhB degradation under visible light irradiation. (**B**) Radical trapping experiments during the photocatalytic degradation of RhB over a-FeOOH/CNNS-800 composite under visible light irradiation.

Recycling experiments were also performed on a-FeOOH/CNNS-800 to evaluate the stability of this photocatalyst (Fig. 11A). Within the same irradiation time (~240 min) more than ~95% RhB were degraded even after seven successive cycles, illustrating its high stability and great promise in practical applications.^48^

The organic pollutants can be effectively degraded by reactive species, including h^+^, ∙OH and ∙O_2_^−^, which may vary for different photocatalysts due to their different band structures and phase compositions^12^. Therefore, to explore the mechanism of the high photocatalytic activity and to assess the contribution of the reactive species, trapping experiments of reactive species were conducted using ethylenediaminetetraacetate (EDTA-2Na) and isopropyle alcohol (IPA) as h^+^ and ∙OH scavenges, respectively^49, 50^. By adding two scavenges into the degradation solutions, the reactive species in the degradation process can be revealed, as illustrated in Fig. 11B for the degradation of RhB by a-FeOOH/CNNS-800. The addition of IPA and EDTA-2Na has a certain effect on the photocatalytic efficiency, indicating that the •OH and h^+^ species play a positive role in the photocatalytic degradation of RhB.

**Figure 12:**
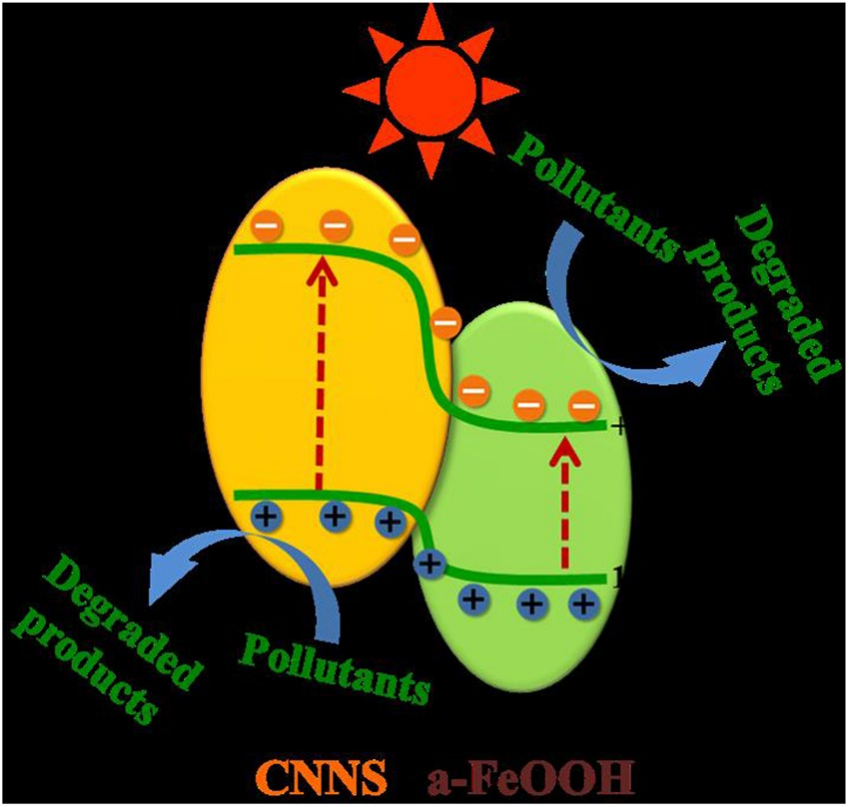
Proposed mechanisms of photo-generated charge transfer and pollutants degradation in the a-FeOOH/CNNS composites under visible light irradiation.

Based on experimental results and previous researches, Fig. 12 depicted a diagrammatic sketch for the energy band positions of CNNS and a-FeOOH^6, 20, 51^. The conduction bands of CNNS and a-FeOOH are −1.38 eV and 0.58 eV^6, 18, 51^, so the valence bands CNNS and a-FeOOH are 1.18 eV and 1.49 eV, respectively. Under visible light irradiation, CNNS and a-FeOOH could absorb visible light, leading to the excitation of e^-^ to the conduction band (CB) and whilst keeping h^+^ in the valence bands (VB). For a-FeOOH/CNNS heterojunctions, the photogenerated e^-^ on the CNNS CB could easily migrate to the CB of a-FeOOH while the photogenerated h^+^ in the VB of a-FeOOH could migrate to CNNS. That is to say, the appropriately aligned band edges of CNNS and a-FeOOH indicated that the migration of effective photogenerated charges could occur via the heterojunctions with strong interfacial coupling effect in the composite. The migration of photogenerated charges limited the transmission of photogenerated e^-^ and h^+^ on different sides, which reduced the recombination rate of photogenerated electron-hole pairs and improved the abundance and stability of photogenerated charge in the composite. At the same time, the isolated photogenerated charge promoted the production of reactive oxidative species, i.e. h^+^ and •OH, which were responsible for degrading RhB. And the conclusion can be further confirmed by Fig. 11B. So the photocatalytic efficiency of the composite was improved.

Generally, higher specific surface areas and larger pore volumes of photocatalysts were favorable for photocatalytic reaction, which can provide abundant reaction sites^3, 50^. The nitrogen adsorption-desorption isotherms and the pore size distributions curves of pure CNNS and a-FeOOH/CNNS-800 are showed in Figure S6. The BET surface areas of pure CNNS and a-FeOOH/CNNS-800 are 90.59 and 90.68 m^2^/g respectively, which means that they have higher BET surface areas. At the same time, the pure CNNS and a-FeOOH/CNNS-800 also have some mesopores. Compared with pure CNNS, a-FeOOH/CNNS-800 has more mesopores, which is consistent with the degradation performance of the photocatalysts. Therefore, the pure CNNS and a-FeOOH/CNNS-800 can provide abundant reaction sites for photocatalytic reactions due to their higher specific surface areas and larger pore volumes.

In the field of photocatalysis, photo absorption induced electron–hole separation character is extremely important^3^. To further comprehend the separation and recombination of electron–hole pairs in pure CNNS and a-FeOOH/CNNS composites, the photocurrent test is carried out under visible light, as illustrated in Figure S7. As we all know, the higher the photocurrent, the higher electrons–holes separation efficiency, thus resulting in higher photocatalytic activity^1–3^. In this study, a-FeOOH/CNNS-800 composite shows the highest photocurrent intensity, which is more than 2 times than that of pure CNNS. This indicates that a-FeOOH/CNNS-800 composite has the lowest electrons and holes recombination rate. Thus we think a-FeOOH/CNNS composites may have very good photocatalyst performance.

In order to study the effect of a-FeOOH modification, photoluminescence (PL) spectral analysis was carried out to bright to light the migration, transfer and recombination processes of photo-generated electron–hole pairs in pure CNNS and a-FeOOH/CNNS composites^2, 11^. As shown in Figure S8, the main fluorescence emission peak is centered at 500 nm for CNNS. With the addition of a-FeOOH nanoparticles, the emission intensity of the PL spectra of a-FeOOH/CNNS composites is significantly lower than that of CNNS and the order of the a-FeOOH/CNNS composites is consistent with the photocatalytic performance. Therefore, the a-FeOOH/CNNS composites inhibit more effectively the recombination of photo-generated charge carriers to improve photocatalytic activity. This give further an evidence to support the above photocurrent response curves results.

## Conclusions

In summary, a series of a-FeOOH/CNNS composites was successfully synthesized via a facile *in-situ* precipitation method. The hierarchically ultrathin g-C_3_N_4_ nanosheets not only provided a large surface area, but also performed as the support to form heterostructures. The as-synthesized a-FeOOH/CNNS-800 photocatalyst showed superior visible light photocatalytic activities than others, which could be ascribed to the synergetic effect between a-FeOOH and CNNS, including the maximum heterojunction interface with intimate contact, enhanced photogenerated charge separation efficiency, and fully exposed reactive sites as well as excellent visible light response in the composite. This work could give insights into the importance of rational design of heterojunction systems, and provide a potential method for the construction of efficient heterojunction photocatalysts with controllable sizes and space distributions.

## Electronic supplementary material

Scientific Rep-Iron oxyhydroxide_g-C3N4 -SI-sub
