# Supplementary material for: Constructing the novel ultrafine amorphous iron oxyhydroxide/g-C3N4 nanosheets heterojunctions for highly improved photocatalytic performance
Source: Sci Rep. 2017 Aug 17;7:8686. doi: 10.1038/s41598-017-09283-1 (PMC5561240; doi:10.1038/s41598-017-09283-1)
Supplement: Supplementary file 1 — Scientific Rep-Iron oxyhydroxide_g-C3N4 -SI-sub [file 41598_2017_9283_MOESM1_ESM.pdf]

Constructing the novel ultrafine amorphous iron oxyhydroxide/g-C_3N_4 nanosheets heterojunctions for highly improved photocatalytic performance

Hongcen Yang, Shouwei Zhang, Ruya Cao, Xiaolong Deng, Zhipeng Li, Xijin Xu**

School of Physics and Technology, University of Jinan, Shandong 250022, PR China

E-mail: sps_xuxj@ujn.edu.cn; zhangsw-1122@163.com

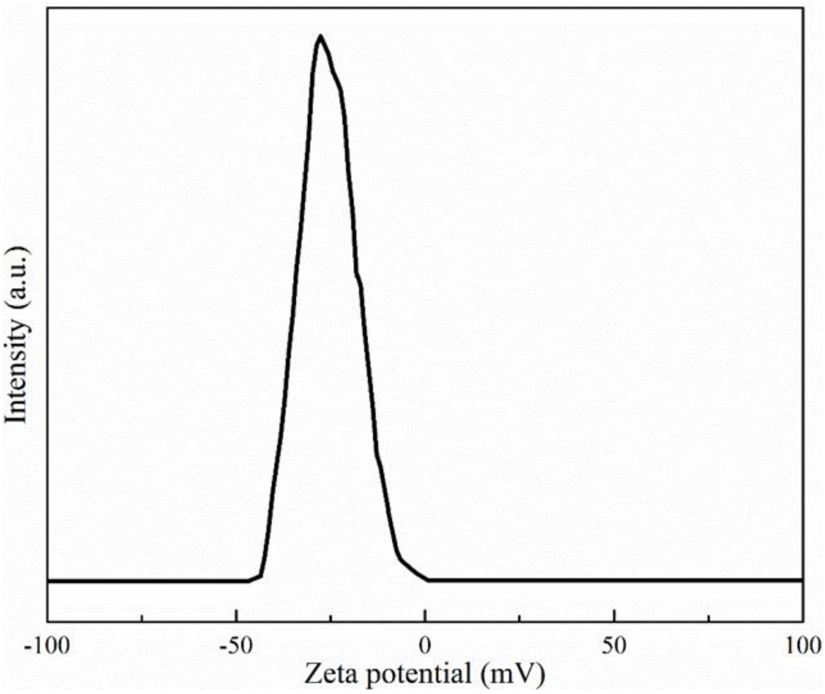

Figure S1. Zeta potential of ultrathin CNNS dispersed in water.

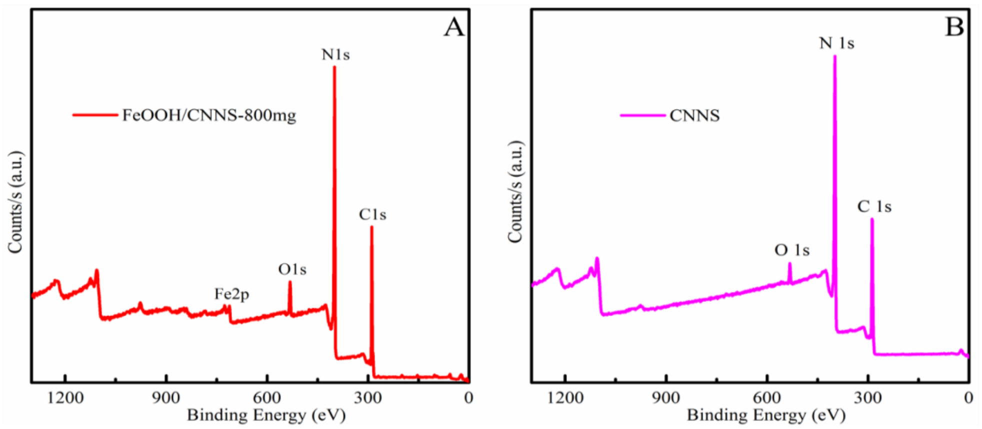

Figure S2. The survey XPS spectra of (A) a-FeOOH/CNNS-500 composite and (B) pure CNNS.

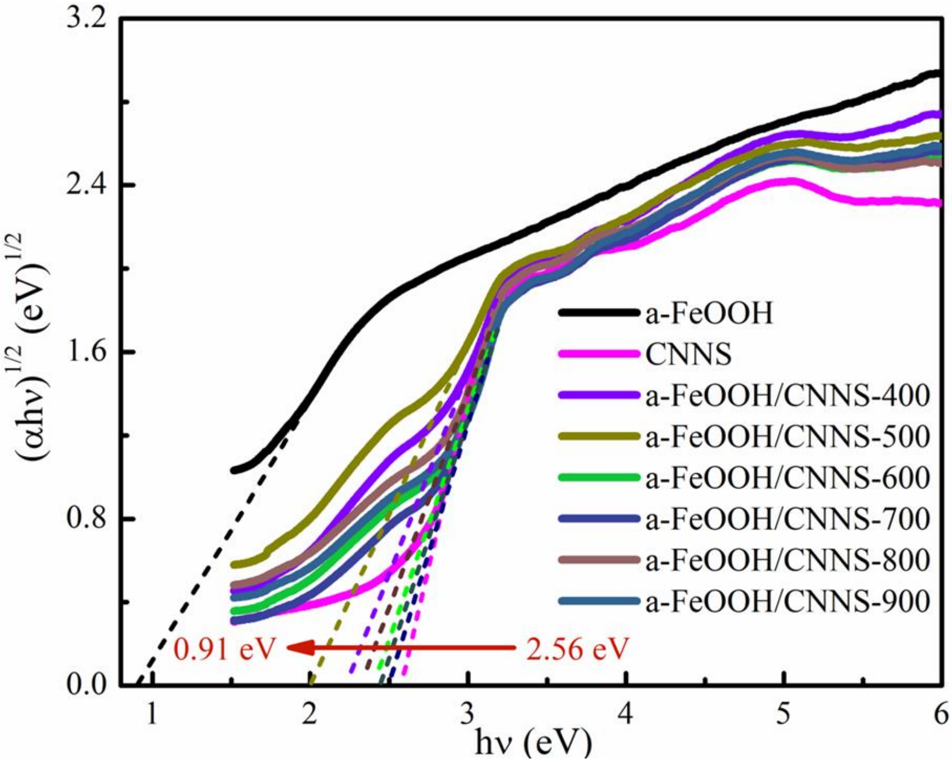

Figure S3. Plot of $(\alpha h\nu)^{1/2}$ vs. hν for the band gap energy of pure CNNS and a-FeOOH/CNNS composites.

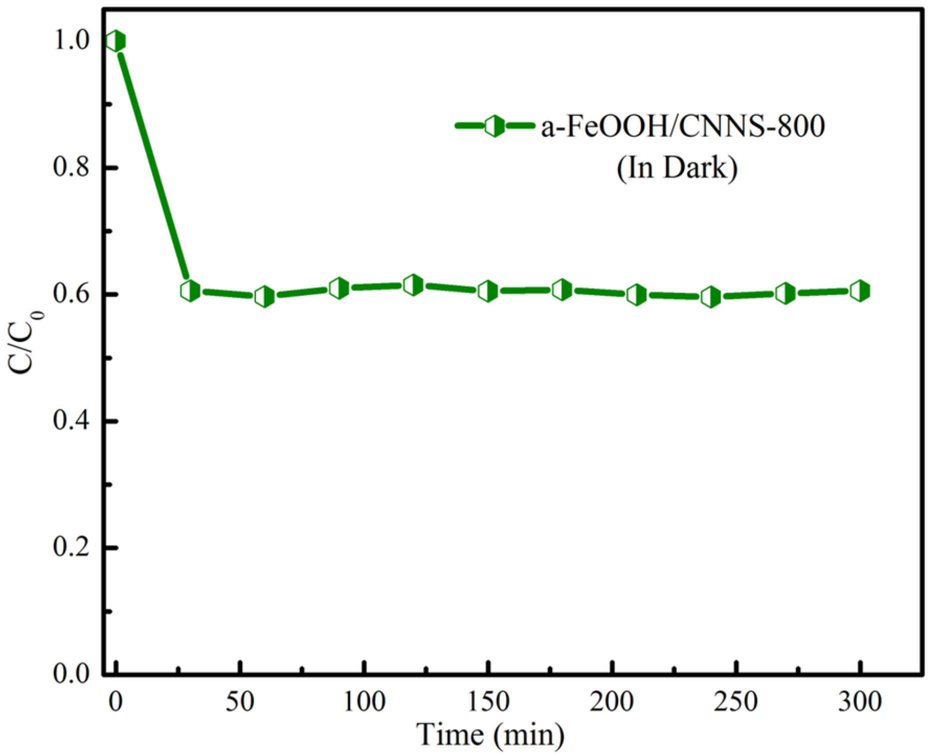

Figure S4. The adsorption activity of the as-prepared photocatalyst for the adsorption of RhB in dark.

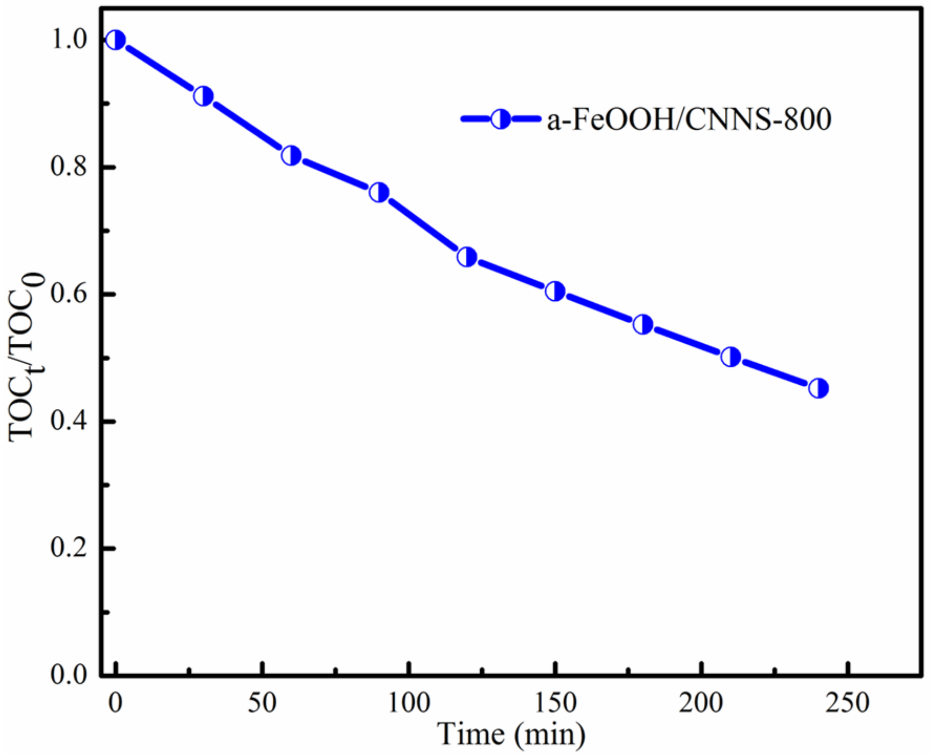

Figure S5. TOC removal during the degradation of RhB.

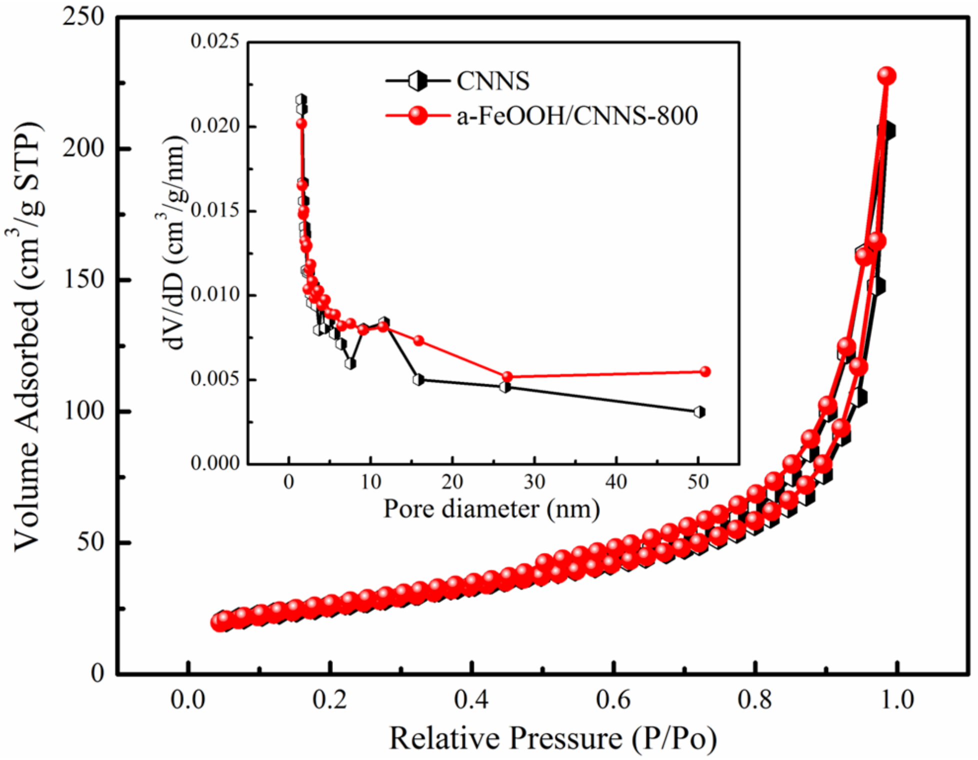

Figure S6. Nitrogen sorption isotherm and Barrett–Joyner–Halenda (BJH) pore size distribution plot (inset) of pure CNNS and a-FeOOH/CNNS-800.

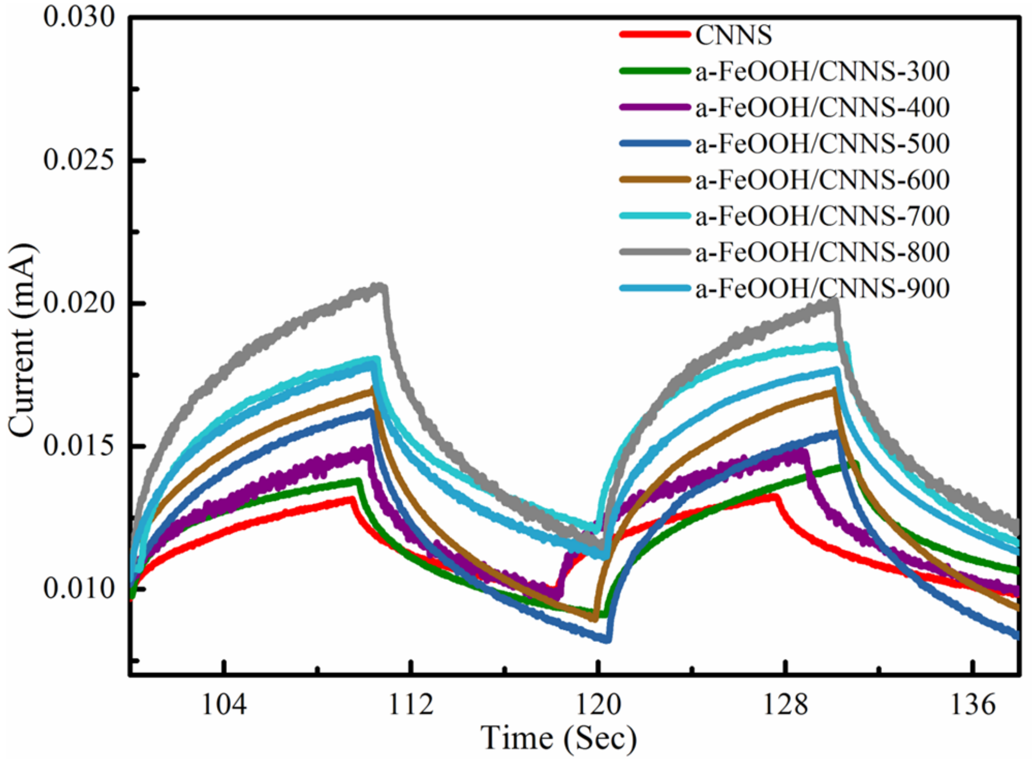

Figure S7. Photocurrent response curves of pure CNNS and a-FeOOH/CNNS composites under visible light irradiation.

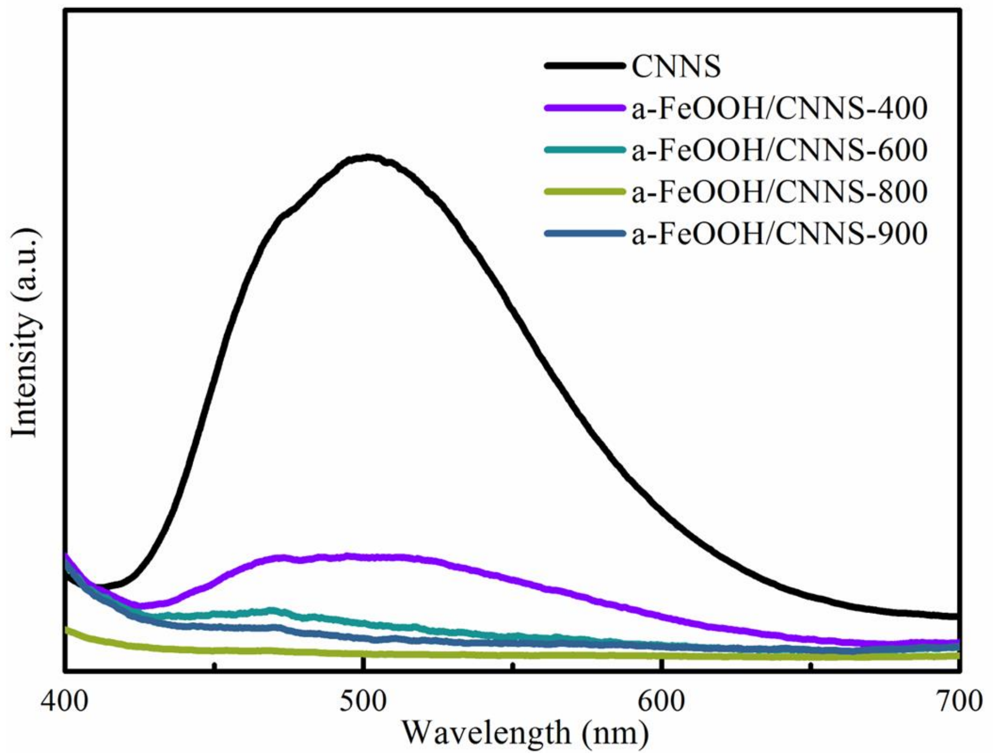

Figure S8. Photoluminescence (PL) spectra of pure CNNS and a-FeOOH/CNNS composites.
